# In Vitro Transdifferentiation Potential of Equine Mesenchymal Stem Cells into Schwann-Like Cells

**DOI:** 10.1089/scd.2022.0274

**Published:** 2023-06-30

**Authors:** Lucas Vinícius de Oliveira Ferreira, Beatriz da Costa Kamura, João Pedro Marmol de Oliveira, Natielly Dias Chimenes, Márcio de Carvalho, Leandro Alves dos Santos, Luciane Alarcão Dias-Melicio, Renée Laufer Amorim, Rogério Martins Amorim

**Affiliations:** ^1^ Department of Veterinary Clinics, School of Veterinary Medicine and Animal Science; São Paulo State University (UNESP), Botucatu, São Paulo, Brazil.; ^2^ Translational Nucleus of Regenerative Medicine (NUTRAMERE), School of Veterinary Medicine and Animal Science; São Paulo State University (UNESP), Botucatu, São Paulo, Brazil.; ^3^ Confocal Microscopy Laboratory, UNIPEX–Experimental Research Unit, Medical School of Botucatu; São Paulo State University (UNESP), Botucatu, São Paulo, Brazil.; ^4^ Department of Pathology, Medical School of Botucatu; São Paulo State University (UNESP), Botucatu, São Paulo, Brazil.

**Keywords:** facial nerve, nerve regeneration, neurotrophic factors, peripheral nerve injury, Schwann-like cells

## Abstract

Schwann cells (SCs) are essential for the regenerative processes of peripheral nerve injuries. However, their use in cell therapy is limited. In this context, several studies have demonstrated the ability of mesenchymal stem cells (MSCs) to transdifferentiate into Schwann-like cells (SLCs) using chemical protocols or co-culture with SCs. Here, we describe for the first time the in vitro transdifferentiation potential of MSCs derived from equine adipose tissue (AT) and equine bone marrow (BM) into SLCs using a practical method. In this study, the facial nerve of a horse was collected, cut into fragments, and incubated in cell culture medium for 48 h. This medium was used to transdifferentiate the MSCs into SLCs. Equine AT-MSCs and BM-MSCs were incubated with the induction medium for 5 days. After this period, the morphology, cell viability, metabolic activity, gene expression of glial markers glial fibrillary acidic protein (GFAP), myelin basic protein (MBP), p75 and S100β, nerve growth factor (NGF), brain-derived neurotrophic factor (BDNF), and glial cell-derived neurotrophic factor (GDNF), and the protein expression of S100 and GFAP were evaluated in undifferentiated and differentiated cells. The MSCs from the two sources incubated with the induction medium exhibited similar morphology to the SCs and maintained cell viability and metabolic activity. There was a significant increase in the gene expression of BDNF, GDNF, GFAP, MBP, p75, and S100β in equine AT-MSCs and GDNF, GFAP, MBP, p75, and S100β in equine BM-MSCs post-differentiation. Immunofluorescence analysis revealed GFAP expression in undifferentiated and differentiated cells, with a significant increase in the integrated pixel density in differentiated cells and S100 was only expressed in differentiated cells from both sources. These findings indicate that equine AT-MSCs and BM-MSCs have great transdifferentiation potential into SLCs using this method, and they represent a promising strategy for cell-based therapy for peripheral nerve regeneration in horses.

## Introduction

Peripheral nervous system (PNS) injuries are common in humans and animals [1,2]. Persistent sensorimotor dysfunction is the main consequence of injury and affects the patient's quality of life [3,4].

The causes of PNS injuries in equine are multiple and can occur due to traumatic processes, and metabolic, toxic, infectious, and degenerative diseases [5,6]. One of the most common neuropathies in horses is facial nerve paralysis, which is associated with traumatic events in most cases [7].

Despite the regenerative potential of the PNS, predominantly attributed to the plasticity of Schwann cells (SCs), spontaneous repair is mostly unsatisfactory [8,9]. Although new treatments have been introduced, functional nerve recovery is associated with a series of limitations [10,11]. Cell-based therapy has attracted interest in regenerative medicine and has been proposed as a promising strategy for the regeneration of injured peripheral nerves [12].

The SCs are fundamental in the regeneration process, as they provide support and guide the growth of axons through the secretion of neurotrophic factors, cytokines, and phagocytosis of myelin debris [13,14].

The SCs transplantation has shown beneficial effects in PNS lesions [15,16]; however, for its acquisition and use in clinical practice, it is necessary to sacrifice a healthy functional nerve from the donor, in addition to the fact that the proliferation of these cells in vitro is associated with a long period of cultivation and expansion [9,17,18].

To overcome these limitations, several studies have demonstrated the ability of mesenchymal stem cells (MSCs) from different sources and species to transdifferentiate in vitro into Schwann-like cells (SLCs), usually through co-culture with SCs or by using chemical protocols [14,19–24]. However, these induction methods have disadvantages such as complicated protocols and high costs [25,26].

Further, a practical approach was successfully described using a culture medium conditioned with neurotrophic factors secreted from the sciatic nerve of rats to induce in vitro transdifferentiation of rat MSCs derived from adipose tissue (AT-MSCs) and bone marrow (BM-MSCs) into SLCs [25–27].

In equine species, a study showed that treatment of BM-MSCs with a combination of chemicals under specific in vitro conditions resulted in their transdifferentiation into SLCs [22]. Equine MSCs are mainly obtained from AT and BM [28,29]. However, AT-MSCs have several advantages, such as ease of isolation, wide availability, and high in vitro proliferation rate [14,30].

To our knowledge, there are no studies describing the ability to transdifferentiate equine AT-MSCs into SLCs in vitro and there are no reports on the development of a conditioned culture medium with factors secreted from the peripheral nerve of horses and its use to perform transdifferentiation of equine MSCs into SLCs.

Therefore, the present study aimed at evaluating the in vitro transdifferentiation potential of equine AT-MSCs and BM-MSCs into SLCs using a conditioned medium obtained from equine facial nerve explant culture to develop cell-based therapies for the regeneration of injured peripheral nerve in horses.

## Materials and Methods

### Animal ethics

All stages of this study were conducted in accordance with the Ethical Principles in Animal Experimentation and were approved by the Ethics Committee for the Use of Animals (CEUA) of São Paulo State University (UNESP), Botucatu, Brazil, according to protocol number 0039/2021.

### Facial nerve collection and preparation of induction medium

An equine female, 3 years old, Mangalarga, 300 kg was euthanized due to musculoskeletal injuries and permanent recumbency. A facial nerve (∼10 cm) from the horse was collected immediately after euthanasia, under aseptic conditions, and placed in a sterile 50 mL conical tube containing Dulbecco's phosphate-buffered saline (DPBS; LGC Biotecnologia, São Paulo, Brazil), 3% penicillin/streptomycin, and 0.5% amphotericin B (both from Gibco, Grand Island, NY).

The procedure for preparing the induction medium and the method for transdifferentiation of equine MSCs into SLCs were carried out according to the methodology first described using rat sciatic nerves and rat AT-MSCs by Liu et al. [25], with modifications. The facial nerve was washed with DPBS containing 1% penicillin/streptomycin. All excess tissue adhered to the nerve was removed, and the nerve was cut into 1 cm fragments within a laminar flow under sterile conditions.

**FIG. 1.**
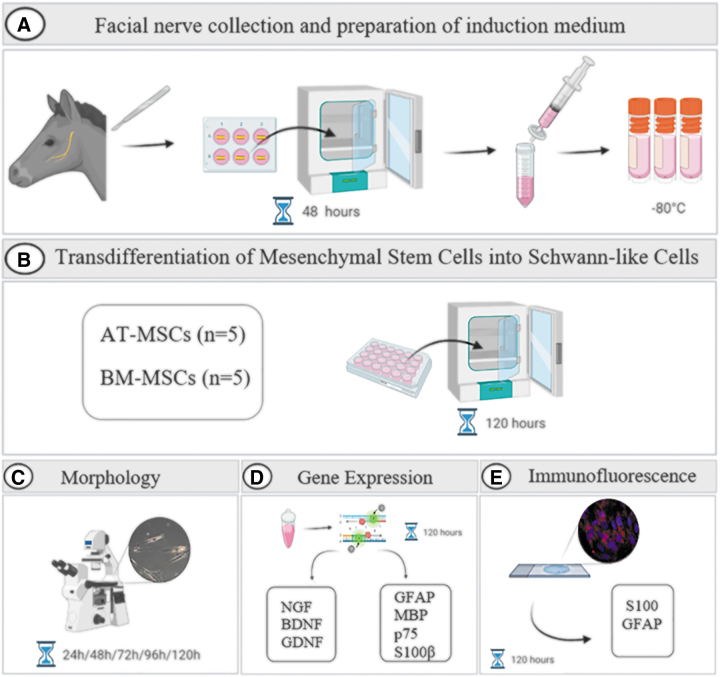
A summary of the experimental design. The facial nerve from the horse was collected immediately after euthanasia. The nerve was cut into 1 cm fragments. Each fragment was soaked in 15 mL of complete culture medium and incubated for 48 h. After this period, the culture medium was aspirated, filtered, and cryopreserved at −80°C **(A)**. Equine AT-MSCs (*n* = 5) and equine BM-MSCs (*n* = 5) were seeded in 24-well plates to perform transdifferentiation into SLCs. Cells were incubated with the induction medium for 120 h **(B)**. The morphology of equine MSCs was evaluated during the period of transdifferentiation using inverted microscopy **(C)**. After this period, the gene expression of glial markers and neurotrophic factors was quantified in undifferentiated and differentiated equine AT-MSCs and BM-MSCs. qPCR was performed **(D)**. Protein expression of glial markers was analyzed using immunofluorescence staining **(E)**. AT, adipose tissue; BM, bone marrow; MSC, mesenchymal stem cell; qPCR, quantitative real-time polymerase chain reaction; SLC, Schwann-like cell. Created with Biorender.com

Each fragment was soaked in 15 mL of complete culture medium containing 90% Dulbecco's modified Eagle's medium (DMEM)/F12 (LGC Biotecnologia), 10% fetal bovine serum (FBS; Gibco), 1% penicillin/streptomycin, and 1% amphotericin B and incubated at 37.5°C in a humidified atmosphere containing 95% air and 5% CO_2_ for 48 h. After this period, the culture medium was aspirated and filtered (70 μm cell strainer, BD Falcon, San Jose, CA) to remove nerve fragments. Subsequently, the induction medium was filtered through a 0.22 μm syringe filter (Sarstedt, Nümbrecht, Germany) and cryopreserved at −80°C for the next steps (Fig. 1A).

### Mesenchymal stem cells

Equine MSCs (P3) derived from the AT (*n* = 5) and BM (*n* = 5) were obtained from the cell bank of our research group. The MSCs have been previously characterized (differentiation into osteogenic, chondrogenic, and adipogenic mesenchymal lineages and evaluation of surface markers). Immunophenotypic analysis performed by flow cytometry revealed high expression of the markers CD44, CD90, and CD105 and absence of expression of the markers CD34 and MHC-II [31].

### Transdifferentiation of MSCs into SLCs

The MSCs were thawed and resuspended in complete culture medium. Equine AT-MSCs and BM-MSCs were seeded at a density of 1 × 10^4^ cells per well in 24-well plates (Kasvi, São José do Pinhais, Brazil) with complete culture medium (0.5 mL) containing 90% DMEM/F12, 10% FBS, 1% penicillin/streptomycin, and 1% amphotericin B.

To perform transdifferentiation into SLCs, the culture medium was removed from the subconfluent MSCs cultures and replaced with pre-warmed induction medium (0.5 mL). Cells were incubated under these conditions for 5 days at 37.5°C in a humidified atmosphere containing 95% air and 5% CO_2_. The induction medium was changed every 2–3 days. Equine AT-MSCs and BM-MSCs were divided into two groups: cells cultured in complete culture medium (undifferentiated cells) and those cultured in induction medium (differentiated cells) (Fig. 1B).

### Morphology evaluation

The morphology of equine MSCs was evaluated using an inverted microscope (LEICA DMIRB, Germany). Photomicrographs of undifferentiated and differentiated cells were captured during the transdifferentiation period (Fig. 1C).

### Cell viability

After the transdifferentiation period (120 h), the viability of undifferentiated and differentiated equine AT-MSCs and BM-MSCs was examined using the exclusion test with 0.4% trypan blue. The MSCs were added in a microcentrifuge tube with 0.4% Trypan Blue and placed in a Neubauer chamber to count (the number of viable, dead, and total cells).

### MTT assay

MTT [3-(4,5-dimethylthiazol-2-yl)-2,5-diphenyltetrazolium bromide] was performed to evaluate the metabolic activity of undifferentiated and differentiated equine MSCs. This assay provides an estimate of the number of metabolically active cells. Undifferentiated and differentiated equine MSCs were seeded in 96-well plates (Sarstedt, Nümbrecht, Germany) at a concentration of 1 × 10^4^ cells per well and incubated at 37.5°C in a humidified atmosphere containing 95% air and 5% CO_2_. After 24 h of cultivation, the supernatant was removed and the MSCs were incubated in an MTT solution for 4 h at 37.5°C and 5% CO_2_.

Subsequently, the MTT solution was removed and the cells were homogenized with 200 μL dimethyl sulfoxide per well. Absorbance values were obtained in the range of 570 nm in a microplate reader (Biochrom Asys Expert Plus Microplate Reader; Biochrom Ltd., Harvard Bioscience, Holliston, MA).

### Gene expression: quantitative real-time polymerase chain reaction

The gene expression of the glial markers such as glial fibrillary acidic protein (*GFAP*), myelin basic protein (*MBP*), *p75* and *S100β*, and the neurotrophic factors brain-derived neurotrophic factor (*BDNF*), glial cell-derived neurotrophic factor (*GDNF*), and nerve growth factor (*NGF*) was quantified in undifferentiated and differentiated cells derived from equine AT-MSCs and BM-MSCs after the transdifferentiation period (120 h) (Fig. 1D).

Cellular RNA was extracted using TRIzol reagent (TRIzol™, Invitrogen™; Thermo Fisher Scientific, São Paulo, Brazil) according to the manufacturer's instructions. The RNA was eluted with RNA-free water, quantified, and the absorbance ratios of 260/280 and 260/230 nm were determined using the Thermo Scientific NanoDrop 2000 spectrophotometer (Thermo Fisher Scientific, Wilmington). Total RNA extracted from the cells was of high quality and purity, indicating that the extraction method was efficient.

cDNA synthesis was performed using the High-Capacity cDNA Reverse Transcription Kit (Applied Biosystems™; Life Technologies Corporation, Carlsbad), according to the manufacturer's instructions, and reverse transcription was performed to obtain cDNA with the Veriti 96 Well Thermal Cycler (Applied Biosystems; Thermo Fisher Scientific), under the following thermal cycling conditions: 10 min at 25°C, 12 min at 37°C, and 5 min at 85°C. The cDNA samples were cryopreserved at −80°C and used as templates for polymerase chain reaction (PCR).

PCR was performed in duplicate using PowerUp™ SYBR™ Green Master Mix (Applied Biosystems; Life Technologies), RNA-free water, and equine primers (Thermo Fisher Scientific), which were designed using Primer Express™ Software v3.0.1 (Applied Biosystems; Thermo Fischer Scientific), as shown in Table 1.

**Table 1.** The Primer Sets Used for Quantitative Polymerase Chain Reaction

Gene	Forward primer	Reverse primer
*BDNF*	TTGGATGAGGGCCAGAAAGT	CAAGTCCGCGTCCTTACTGTT
*GDNF*	CAGGGACTCTTCCTCCATCCT	TGGGCACGAGCATGTTTCT
*NGF*	CCAACGGAGCAGCTTTCTGT	AACAACATGGACATTACGCTATGC
*GFAP*	GAAACCAGCCTGGACACCAA	TCACCACGATGTTCCTCTTGAG
*MBP*	TGTACGAGGCGTCACAATGTTC	GGCCCCAAGACGAAAACC
*p75*	GAAGAGCCGGCCTTAGAGTGT	CCTCTGTAGGGTCCGCTAAACC
*S100β*	CGCTCATGGCTATTGGTGAA	TCGTGGATACAAGAGCAAAAGTT
*ACTB*	CGGCGGCTCCATTCTG	CTGCTTGCTGATCCACATCTG
*B2M*	CACCCAGCAGAGAATGGAAAG	CGGATGGAACCCAGAGACA
*GAPDH*	GGCAAGTTCCATGGCACAGT	GGGCTTTCCGTTGATGACAA
*HPRT*	GCTCGAGATGTGATGAAGGAGAT	CCCCCTTGAGCACACAGAGT

ACTB, beta-actin; B2M, beta-2-microglobulin; BDNF, brain-derived neurotrophic factor; GAPDH, glyceraldehyde 3-phosphate dehydrogenase; GDNF, glial cell-derived neurotrophic factor; GFAP, glial fibrillary acidic protein; HPRT, hypoxanthine-guanine phosphoribosyltransferase; MBP, myelin basic protein; NGF, nerve growth factor.

The samples were tested with four reference genes, beta-actin (*ACTB*), beta-2-microglobulin (*B2M*), glyceraldehyde 3-phosphate dehydrogenase (*GAPDH*), and hypoxanthine-guanine phosphoribosyltransferase (*HPRT*). The quantitative real-time polymerase chain reaction (qPCR) method was performed using the QuantStudio™ 12K Flex Real-Time PCR System thermocycler (Applied Biosystems; Thermo Fischer Scientific) with the following parameters: 50°C for 2 min, 95°C for 2 min, and 40 cycles of 95°C for 1 s and 60°C for 30 min followed by a dissociation curve. Relative quantification of the expression of genes of interest was performed using the ΔΔCt method [32].

### Immunofluorescence

The protein expression of glial markers was determined using immunofluorescence staining (Fig. 1E). Briefly, equine AT-MSCs and BM-MSCs were seeded at a density of 1 × 10^4^ cells/cm^2^ on 13 mm round coverslips (Kasvi) previously treated with poly-l-lysine (Sigma-Aldrich^®^, Saint Louis, MO) in an adherent 24-well plate (Kasvi) and the transdifferentiation process was performed.

After the transdifferentiation period, undifferentiated and differentiated equine MSCs were fixed with 2% paraformaldehyde in phosphate-buffered saline (PBS) for 20 min, permeabilized with 0.5% Triton X-100 for 10 min at room temperature, and blocked with 2% bovine serum albumin for 20 min (all from Sigma-Aldrich).

Cells were incubated overnight at 4°C with antibodies against S100 (polyclonal rabbit anti-S100, DAKO^®^ GA504, 1:200) and GFAP (polyclonal rabbit anti-GFAP, DAKO GA524, 1:200). Subsequently, the cells were washed with PBS and incubated with goat anti-rabbit IgG H&L (Alexa Fluor^®^ 594) (ab150080; Abcam) secondary antibody for 1 h at room temperature. Vectashield mounting medium with DAPI (Vector Laboratories, Burlingame, CA) was used for mounting.

For the qualitative analysis of S100 and GFAP immunofluorescence, confocal images of undifferentiated and differentiated cells were taken using a LEICA TCS SP8 confocal microscope using LAS X software.

Each slide was analyzed in three different fields, and the images were imported to determinate the integrated pixel density that represented the intensity of labeling using the ImageJ software (version 1.33u; National Institutes of Health). For this purpose, images were always taken using the same set of acquisitions.

### Statistical analysis

Cell viability, cellular metabolic activity, relative quantification, and integrated pixel density were assessed for normality using statistical tests (Shapiro–Wilk), descriptive statistics, and graphic analysis. For parametric data, *t*-test was performed using unpaired samples. For non-parametric data, the Mann–Whitney test was performed for unpaired samples. The level of significance between groups was set at *P* < 0.05. Differences are denoted by a single asterisk (*P* < 0.05) (GraphPad Prism version 8 for Mac, San Diego).

## Results

### Morphological changes

**FIG. 2.**
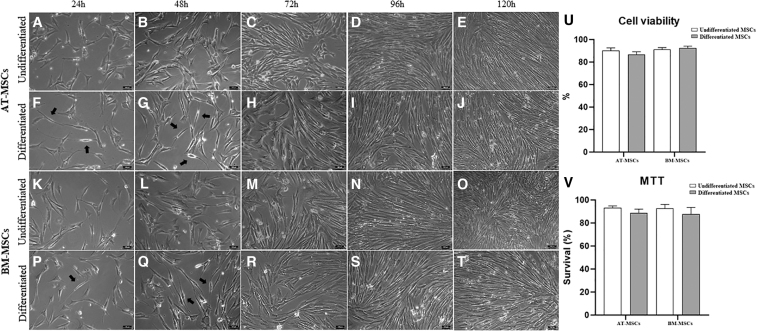
Characterization of morphological differentiation, viability, and metabolic activity of equine MSCs. Undifferentiated equine AT-MSCs and BM-MSCs at 24, 48, 72, 96, and 120 h of culture **(A–E, K–O**, respectively**)** showing fibroblastoid morphology without cytoplasmic extensions. Differentiated equine AT-MSCs and BM-MSCs at 24, 48, 72, 96, 120 h after transdifferentiation induction **(F–J**, **P–T**, respectively**)**. Morphological changes were more evident in areas with lower cell density in equine AT-MSCs and BM-MSCs at 24 and 48 h after transdifferentiation induction **(F, G, P, Q,** respectively**)**; cells showed a fusiform bipolar or tripolar morphology with large nuclei and elongated, thin cytoplasm (*black arrows*). Magnification, 200 × . Scale bar = 100 μm. Cell viability of undifferentiated and differentiated equine AT-MSCs and BM-MSCs **(U)**. Data are represented as the mean ± standard deviation. Metabolic activity of undifferentiated and differentiated equine AT-MSCs and BM-MSCs **(V)**. Data are represented as the mean ± standard deviation.

Fusiform bipolar cells began to appear 24 h after transdifferentiation induction. Cells exhibited bipolar or tripolar morphology with large nuclei, and the cytoplasmic processes became thin and elongated compared with undifferentiated equine AT-MSCs and equine BM-MSCs during incubation with the induction medium (Fig. 2A–T).

### Cell viability

Using the trypan blue exclusion test, we observed cell viability means of 86.8% and 92.4% in the differentiated equine AT-MSCs and BM-MSCs, respectively (Fig. 2U).

### 3-(4,5-Dimethylthiazol-2-yl)-2,5-diphenyltetrazolium bromide

In the evaluation of the metabolic activity of the equine MSCs, there was no significant difference between the groups (undifferentiated and differentiated). We observed metabolic activity means of 88.7% and 87.7% in the differentiated equine AT-MSCs and BM-MSCs, respectively (Fig. 2V).

### Gene expression of glial markers and neurotrophic factors

**FIG. 3.**
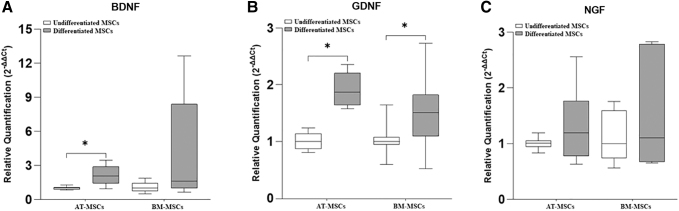
Relative expression of the neurotrophic factors, BDNF, GDNF, and NGF in undifferentiated and differentiated equine AT-MSCs and BM-MSCs. BDNF **(A)**, GDNF **(B)**, NGF **(C)**. Data are represented as medians, interquartile ranges, and minimum and maximum values (*P < 0.05). BDNF, brain-derived neurotrophic factor; GDNF, glial cell-derived neurotrophic factor; NGF, nerve growth factor.

**FIG. 4.**
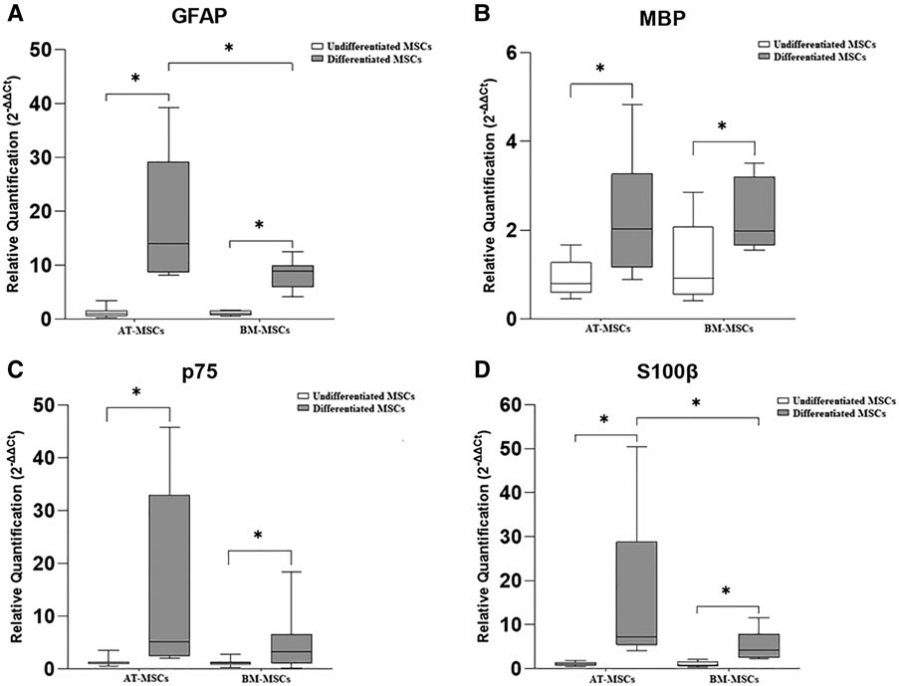
Relative expression of glial markers in undifferentiated and differentiated equine AT-MSCs and BM-MSCs. GFAP **(A)**, MBP **(B)**, p75 **(C)**, and S100β **(D)**. Data are represented as medians, interquartile ranges, and minimum and maximum values (*P < 0.05). GFAP, glial fibrillary acidic protein; MBP, myelin basic protein.

The gene expression of the neurotrophic factors, BDNF, GDNF, and NGF is represented in Fig. 3. and that of the glial markers GFAP, MBP, p75, and S100β is represented in Fig. 4.

In equine AT-MSCs, there was a significant increase in the gene expression of BDNF, GDNF, GFAP, MBP, p75, and S100β in differentiated cells compared with that in undifferentiated cells.

In equine BM-MSCs, there was a significant difference in the gene expression of GDNF, GFAP, MBP, p75, and S100β, which was greater in differentiated cells than in undifferentiated cells. Further, in two samples of equine BM-MSCs, the expression of S100β was observed only in the differentiated cells, whereas in the other three samples, this marker was expressed in both groups (undifferentiated and differentiated) and was higher in the differentiated cells.

Further, the expression of glial markers GFAP and S100β was significantly higher in differentiated AT-MSCs than in differentiated BM-MSCs.

### Immunofluorescence

**FIG. 5.**
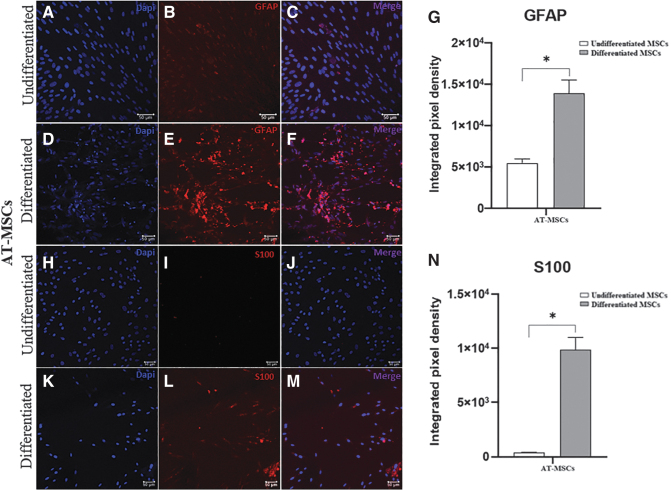
Immunofluorescence staining for glial markers in undifferentiated and differentiated equine AT-MSCs. Representative images of labeling with DAPI (*blue*) and anti-GFAP antibody (*red*) in undifferentiated **(A–C)**, differentiated **(D–F)** AT-MSCs, and integrated pixel density analysis **(G)**. Representative images of labeling with DAPI (*blue*) and anti-S100 antibody (*red*) in undifferentiated **(H–J)**, differentiated **(K–M)** AT-MSCs, and integrated pixel density analysis **(N)**. The glial marker S100 was expressed only in differentiated AT-MSCs. The values of the integrated pixel density are represented as the mean ± standard deviation (*P < 0.05). Scale bar: 50 μm.

**FIG. 6.**
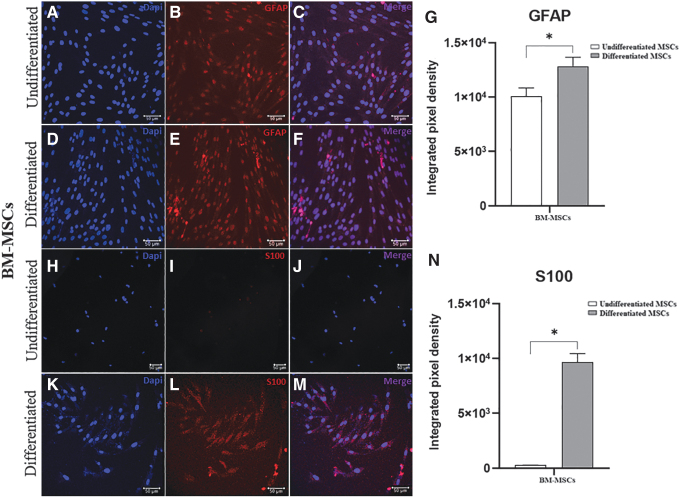
Immunofluorescence staining for glial markers in undifferentiated and differentiated equine BM-MSCs. Representative images of labeling with DAPI (*blue*) and anti-GFAP antibody (*red*) in undifferentiated **(A–C)** and differentiated **(D–F)** BM-MSCs and integrated pixel density analysis **(G)**. Representative images of labeling with DAPI (*blue*) and anti-S100 antibody (*red*) in undifferentiated **(H–J)** and differentiated **(K–M)** BM-MSCs and integrated pixel density analysis **(N)**. The glial marker S100 was expressed only in differentiated BM-MSCs. The values of the integrated pixel density are represented as the mean ± standard deviation (*P < 0.05). Scale bar: 50 μm.

To analyze the transdifferentiation process, we performed immunostaining for GFAP and S100 in equine AT-MSCs and BM-MSCs, as shown in Figs. 5 and 6, respectively. Immunofluorescence staining revealed GFAP expression in undifferentiated and differentiated cells, with a significant increase in the integrated pixel density in differentiated AT-MSCs and BM-MSCs. We observed that the glial marker S100 was expressed in differentiated cells from both sources.

## Discussion

Cell-based therapies such as SCs transplantation are promising for the treatment of peripheral nerve injuries [16]. The SCs are the main glial cells in the PNS and play a crucial role in nerve regeneration after injury [13]. However, two important issues need to be addressed in SCs transplantation in humans and animals. First, the source of the SCs is limited. Second, although extraction and purification of autologous SCs are possible, obtaining SCs in sufficient quantity and of good quality for efficacy is limited [9,17,18]. To solve these issues, previous studies have examined the ability of MSCs to transdifferentiate into glial cells [22,33].

Dezawa et al. [34] described the first protocol to successfully transdifferentiate rat BM-MSCs into SLCs using chemical methods. Based on this transdifferentiation protocol, several modifications have been developed over time using MSCs from different sources and species, such as rats, humans, and equines [14,22,35,36]. However, these transdifferentiation procedures are usually complicated and costly [25,26].

In the present study, we used a conditioned medium obtained from equine facial nerve explant culture to perform the transdifferentiation of equine MSCs based on the protocol performed in rats by Liu et al. [25]. After peripheral nerve injury, several neurotrophic factors, such as BDNF, GDNF, NGF, and neurotrophin-3, are synthesized and secreted by SCs to promote neuroregeneration [10,14,37,38]. In this context, we simulated peripheral nerve injury by sectioning the equine facial nerve into small fragments and incubated them in culture medium for 2 days to obtain conditioned medium containing factors secreted from the nerve.

The methodology used in this study may be applicable due to the possibility of collecting peripheral nerves without injuries to perform the conditioned medium in equine hospitals after death or euthanasia procedures due to the general condition of the animals. Further, equine peripheral nerves can be widely obtained from slaughterhouses.

After incubation with the induction medium, equine AT-MSCs and BM-MSCs showed morphologies similar to those of SCs. Our results agree with those of other studies that have used conditioned medium with secreted factors from the sciatic nerve of rats for in vitro transdifferentiation of MSCs into SLCs [25–27] as well as with studies that have submitted MSCs, derived from different sources and species to chemical protocols [22,39,40].

The SCs secrete several neurotrophic factors that are important molecules that act synergistically to stimulate their proliferation and promote neuronal survival and axonal regeneration [41]. Due to the importance of neurotrophic factors in the regeneration of the PNS, we analyzed the gene expression of BDNF, GDNF, and NGF. We observed differential gene expression of neurotrophic factors in the two sources of equine MSCs after transdifferentiation. Differentiated equine AT-MSCs showed a significant increase in BDNF and GDNF expression, similar to other studies on human AT-MSCs [42,43], whereas differentiated equine BM-MSCs showed only a significant increase in the gene expression of GDNF.

Regarding NGF, we did not observe a significant difference in the gene expression in differentiated cells derived from equine AT-MSCs and BM-MSCs. These results are in agreement with those of Faroni et al. [43] in their study with human AT-MSCs after 18 days of transdifferentiation. Some studies have reported that these results may be attributed to the time of analysis after submitting the cells to different stimuli [43,44].

We examined the gene expression of the glial markers in all equine samples of equine AT-MSCs and BM-MSCs from both groups (undifferentiated and differentiated). Our results demonstrated a significant increase in the expression of all markers analyzed in differentiated cells, corroborating with other experiments carried out with human and rat MSCs derived from different sources [24,36,39]. Comparing the sources of equine MSCs subjected to the induction medium, we observed that the gene expression of GFAP and S100β was significantly higher in equine AT-MSCs, which is the best candidate for cell-based therapy in the context of peripheral nerve injuries.

Our results obtained by immunofluorescence staining were similar to those of another study with equine BM-MSCs, in which GFAP protein was found to be expressed in undifferentiated and differentiated cells and S100 only in SLCs [22]. GFAP protein expression in undifferentiated equine MSCs has already been demonstrated by our study group (unpublished data) and is in agreement with another study on equine BM-MSCs [6].

Previous studies using conditioned medium with factors secreted from the rat sciatic nerve to carry out the process of transdifferentiation of rat AT-MSCs and BM-MSCs into SLCs are in agreement with our data regarding the protein expression of S100 only in differentiated cells, but differed in relation to GFAP expression, as they did not observe the expression of this glial marker in undifferentiated cells but, only in SLCs [25–27].

Most undifferentiated equine MSCs from the two sources used in this study expressed glial markers, without being subjected to any type of induction, suggesting their potential for differentiation into extra-mesodermal lineages [22]. Similar findings have been previously reported [22,40,45–48].

The expression and/or increase in glial markers in differentiated equine MSCs demonstrated the ability of AT-MSCs and BM-MSCs to transdifferentiate into SLCs. This is a promising strategy for obtaining these cells for cell-based therapy of peripheral nerve injuries in horses. Further, after the transdifferentiation process, the differentiated equine AT-MSCs and BM-MSCs maintained high cell viability. This result differs from a previous study using a chemical method to transdifferentiate equine BM-MSCs into SLCs that obtained low viability of differentiated cells [22].

The in vitro transdifferentiation success in this study was determined using morphological analysis and assaying for the expression of SCs markers. Our data corroborate those of several studies performed with MSCs derived from AT and BM of rats, humans, rabbits, and horses submitted to different protocols of transdifferentiation into SLCs [21–23,25,26,39,49].

The expression of glial markers is considered the most relevant indicator to confirm the transdifferentiation of MSCs into SLCs, and although there is no consensus on its use [40,50], GFAP, p75, and S100 markers are commonly used to characterize in vitro transdifferentiation [8,22,23,25–27,39,51,52]. In addition, MBP is synthesized by SC and is associated with myelination, being a vital component of the myelin sheath [53,54].

To the best of our knowledge, the present study is the first to use conditioned medium with factors secreted by the equine peripheral nerve to transdifferentiate equine MSCs into SLCs. The lack of protein identification of the glial markers in differentiated equine MSCs using the western blotting technique and the lack of identification of which factors secreted by the equine facial nerve were fundamental to promote transdifferentiation are the limitations of this study.

We demonstrated the potential of equine AT-MSCs and BM-MSCs to transdifferentiation into SLCs in vitro, and further studies should be carried out to explore the functionality and homing of these SLCs, in addition to the ability to develop 3D-printed nerve guidance conduits functionalized with equine MSCs-derived SLCs for peripheral nerve tissue engineering.

## Conclusion

The approach using conditioned medium obtained from equine facial nerve explant culture altered the morphology and upregulated the gene expression of neurotrophic factors and glial markers in equine AT-MSCs and BM-MSCs, as well as the expression and/or increased glial markers protein expression. Based on our results, we conclude that equine AT-MSCs and BM-MSCs have great transdifferentiation potential into SLCs using conditioned medium obtained from equine facial nerve explant culture, and this is a promising strategy for cell-based therapy for peripheral nerve regeneration in horses.

Further studies should be carried out for exploring the functionality and homing of these SLCs in vivo, providing experimental support for their application in the clinical repair of peripheral nerve injuries.

## Data Availability

The datasets used and/or analyzed during the current study are available from the corresponding author on reasonable request.
